# The first record of exceptionally-preserved spiral coprolites from the Tsagan-Tsab formation (lower cretaceous), Tatal, western Mongolia

**DOI:** 10.1038/s41598-021-87090-5

**Published:** 2021-04-12

**Authors:** Paul Rummy, Kazim Halaclar, He Chen

**Affiliations:** 1 Key Laboratory of Vertebrate Evolution and Human Origins, Institute of Vertebrate Paleontology and Paleoanthropology, Chinese Academy of Sciences, Beijing, 100044 China; 2 CAS Center for Excellence in Life and Paleoenvironment, Beijing, 100044 China; 3 College of Earth and Planetary Sciences, University of Chinese Academy of Sciences, Beijing, 100049 China

**Keywords:** Palaeontology, Palaeoecology

## Abstract

In this paper, seven coprolites from the Lower Cretaceous of Tsagan-Tsab formation have been described. Thus, producing a significant contribution to what we perceived as the first detailed study of coprolites from the Mesozoic deposits in Mongolia. The collected coprolites encompass a total of six spiral amphipolar and one scroll coprolites. We prominently identified four new coprolite ichnotaxa, such as: *Hyronocoprus tsagantsabensis* and *Hyronocoprus hunti*, to which both are ichnosp. nov.; followed by *Megakalocoprus barremianensis* and *Scrollocoprus tatalensis*, where both are ichnogen. et ichnosp. nov. Notably, CT scans revealed that all specimens showed various amounts of bony inclusions and scales, hence, deducing that the producers could have had a low acidic digestive track and were unable to dissolve bone matters. Moreover, SEM–EDS analysis concluded its carnivorous nature, thus, pointing towards piscivorous diet. The small sized *Scrollocoprus* is considered to be the second findings of Mesozoic era’s scroll coprolites, which contain possible plant pollens, a complete infraorbital bone, clusters of bone fragments and rhomboidal-shaped ganoid scales of the prey; and bioerosional scars have been observed on the surface. We suggest those amphipolar spiral ichnotaxa were produced by Asipenceriformes, with Pholidophoriformes as the prey, while *Scrollocoprus* represents fecal excrement of underived fish, possibly of sarcopterygian origins.

## Introduction

Studies on animal fecal excrement can be traced back to the earliest description by Lister in 1678^1^. Duffin^2^ mentioned that the earliest report of vertebrate coprolite could have been written by Edward Lhywd in 1570. Since then, the interest on the subject matter has increased over the years and progressed into the findings of fossilized feces. Coprolite was first termed by Buckland based on fossils uncovered by Mary Anning’s in Lyme Regis, Dorset, southern England. Buckland identified them as petrified fecal excrement that belonged to ichthyosaurs. Previously, those fossils were thought to be fossilized fir cones due to their similar spiral markings, but later on it was suggested to have belonged to animal origins^3,4^. Although Buckland successfully referred the coprolites to marine animals, subsequent studies revealed that those spiral coprolites might have belonged to sharks, rather than what he thought was ichthyosaurs, which were commonly found in Lyme Regis^5,6^.

Coprolite studies have been well known over the past years, and it has become one of the most important research in the subject of trace fossils, since they displayed a wide range of morphological variation, including those that are spiral. According to Hunt & Lucas^7^′s definition, spiral coprolites possess an external appearance that looks like a ribbon, which coils around a long axis, but internally they were formed with piled and spiraling cones. On the other hand, those identified scroll coprolites have a similar structure as to of a roll sheet of paper. Spiral and the scroll morphology are largely influenced by the architecture of the valvular intestine, which are *valvula spiralis* or transverse intestine, followed by *valvula voluta* or longitudinal intestine^8–10^. Some of the earliest studies account on spiral valves in extant fishes were conducted by various scientists since 1667^9,11–15^. As to date, the oldest known spiral coprolites have been recorded from the Soom Shale Lagerstätte of the Upper Ordovician of South Africa^16^, while those of scroll coprolites have been recorded as earliest from the Silurian of Louisburgh, Co. Mayo, Ireland^17^. Generally, the records of scroll coprolite are scarce^7,17^, as they are more commonly known from the Palaeozoic and Cenozoic periods. The first recorded Mesozoic scroll coprolites were known from the Tiki Formation, India^18^.

Spiral coprolites were initially differentiated into two distinct morphotypes, which are the amphipolar and the heteropolar^5^. In subsequent studies, heteropolar edge and knot morphotypes were introduced^19^. With that, it was generally agreed upon that spiral coprolites are indeed the product of animals with a complex spiral valve intestine, such as the sharks, rays, lungfishes and maybe ichthyosaurs^4,20–23^; which are closely associated to aquatic environment; and in generally, rapidly buried.

**Figure 1:**
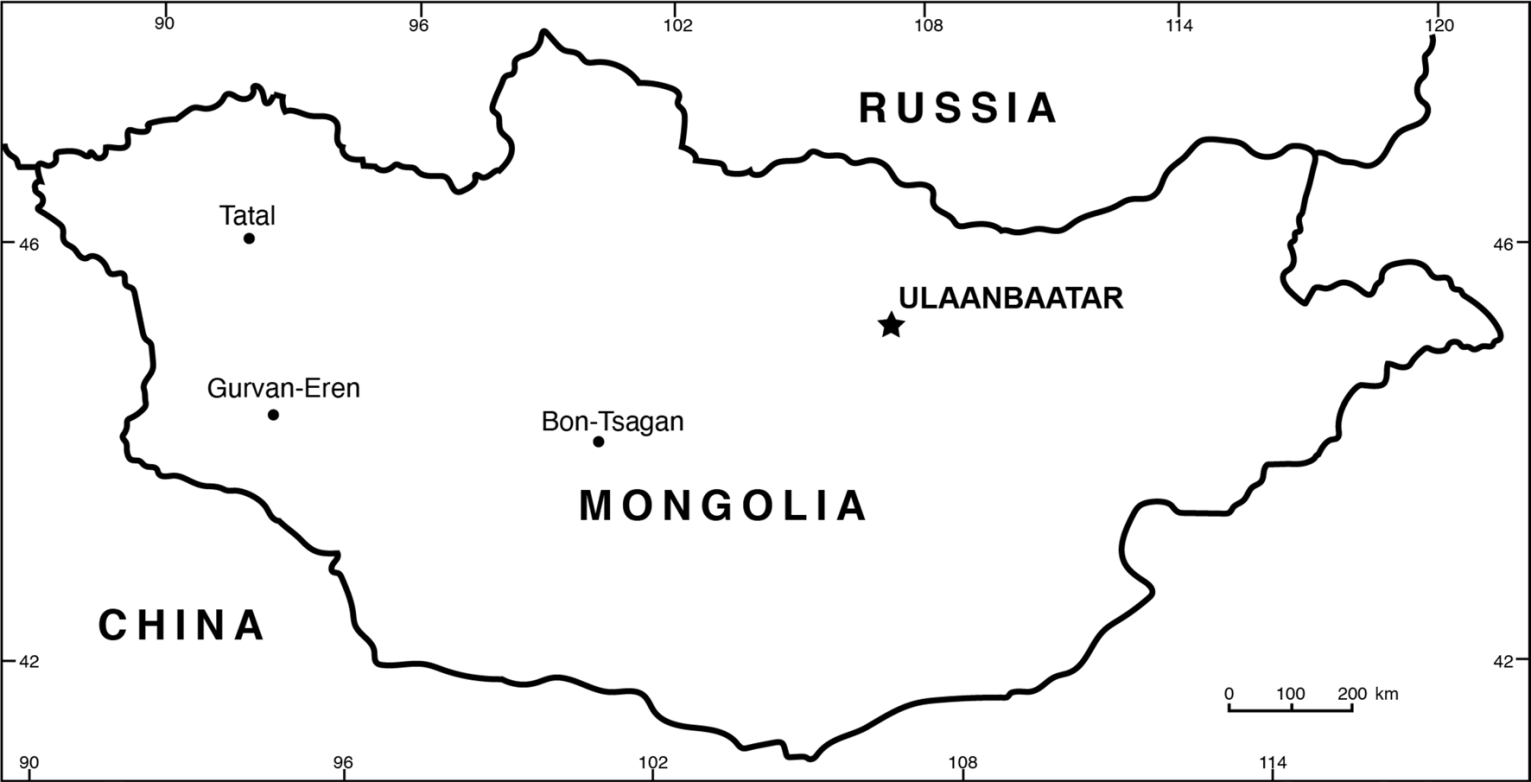
Map showing the location of Tatal, in Mongolia. The coprolites were retrieved from the Tsagan-Tsab formation.

Notably, the significance of coprolite in the studies of paleontology and its contribution to the understanding of ancient ecosystems have been inevitably recognised in recent years. Coprolites from worldwide Phenerozoic have become one of the most important tools in retrieving paleobiological information^24–27^. Furthermore, coprolites did play an important role in preserving the traits of behavior^28^ and it has been acknowledged that coprolites can provide salient analytical diagnostic on the feeding habits and dietary, prey-predator interactions, digestive physiology, diversity of the biota and environment, in which the organism lived to a certain extent on bacterial residues and DNA fragments^29–34^. This paper describes the biogenic structures, which herein attributed mostly to amphipolar spiral coprolites, found in the Lower Cretaceous of Tsagan-Tsab formation, western Mongolia. Also, it is considerably the first detailed study of coprolite from Mongolia. Jakovlev^35^ mentioned the findings of spiral coprolites from the Lower Cretaceous locality of Gurvan-Ereniy-Nuru in West Mongolia (Fig. 1). At that time, they believed that the coprolites could have been produced by Choristoderans. Despite the strong morphological structures of the Tatal’s coprolites, which tends to support its animal feces origins, our study has also discussed on the coloration, composition of the specimen, surface texture, traces of coprophagous organisms and as well as the inclusions within it. A detailed descriptions of the specimens, followed by the succeeding comparison of the anatomical of the intestine features on related extant fishes based on past literatures have narrowed down the potential producer of the amphipolar coprolites, which could potentially belong to Asipenceriformes (sturgeon and paddlefish) (see Discussion and interpretation).

## Geological settings

Tsagan-Tsab/Tsagaantsav/Tsagantsab formation is part of the Basin of Great lakes and Dornogobi basin, and stretches from northwest to southeast of Mongolia^36^. The exact age for this formation is controversial. According to existing literature^37–44^, the Tsagan-Tsab formation could have range from the late Jurassic to Early Cretaceous, while Graham et al.^45^, reported 40Ar/39Ar age of 131 ± 1 Ma (Khara Khutul section) and 126 ± 1 Ma (Tsagan Tsav section), thus suggesting a Hauterivian-Barremian age. Meanwhile, Krassilov^46^ reported a Valanginian to Barrremian age by using plant fossils. It has also been noted in Hasegawa et al.^47^ that the overall climate during this period was dry, due to the presence of reddish beds with calcretes and possible occurrences of intermittent humid climate, because of perennial lacustrine bodies. The Tsagan-Tsab formation is almost 1000 m in thickness, to which it is divided into upper and lower part, and consist of basal conglomerate to trough cross-bedded, coarse- to medium-grained sandstone, reddish or greenish shale, and calcretes^41,47,48^. It mainly consists of alluvial fan to lacustrine deposits^47^ and forms a large shallow lake^36^.

Over the years, Tsagan-Tsab formation has yielded numerous fossil fauna and among some, but not limited, include insects, mollusca, ostracods, fish, lizards, pterosaurs and indeterminate psittacosaurid, sauropods and theropods^36,42,44,49,50^.

The specimens were excavated at Tatal, western Mongolia (Fig. 1).

## Systematic Ichnology

**Hyronocoprus, Hunt et al., 2005**

*Hyronocoprus tsagantsabensis*, ichnosp. nov., Rummy et al., 2021.

**Figure 2:**
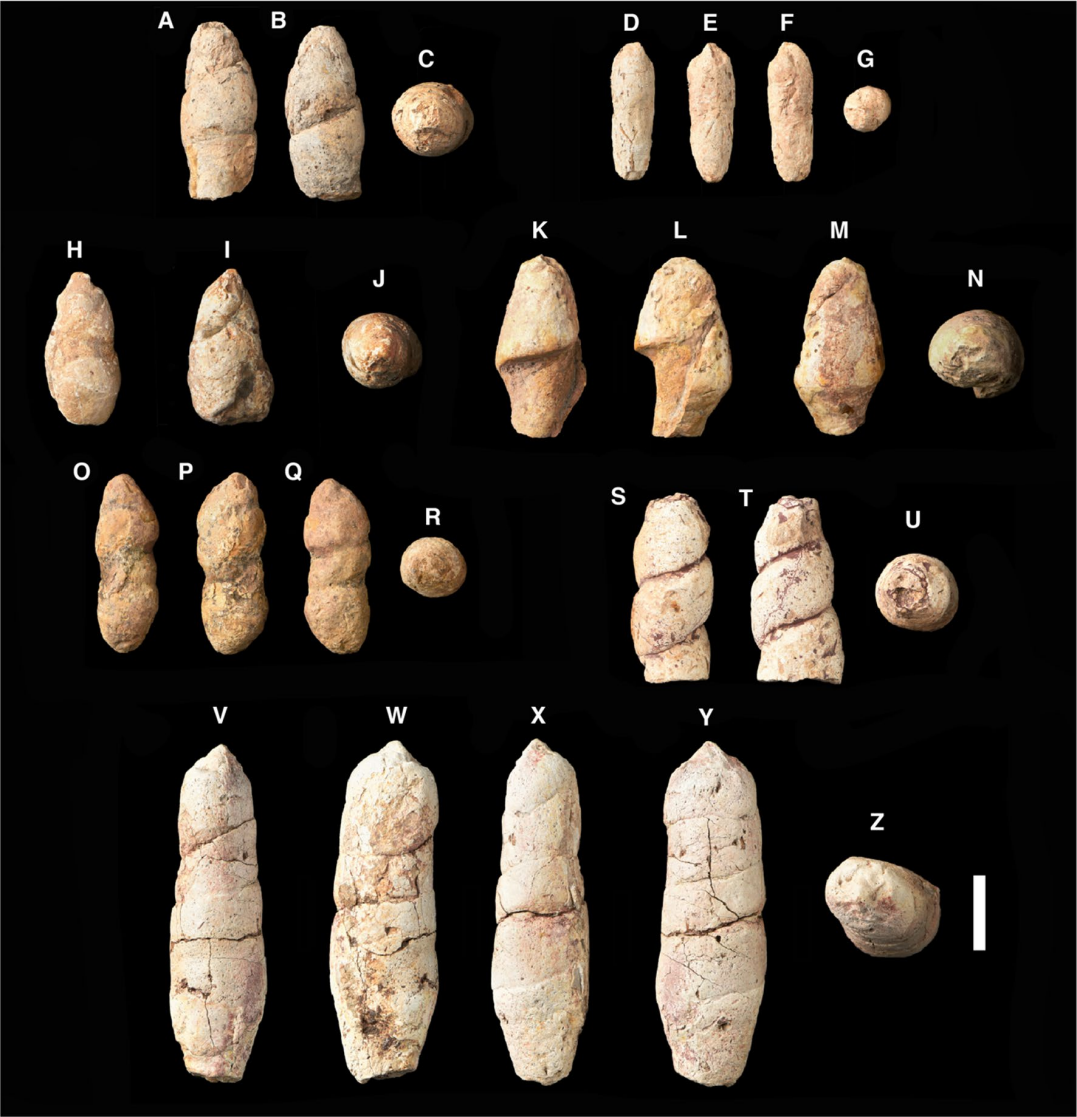
Coprolites from Tatal. (A—B) Specimen IVPP V 27,544 in different views. (D-G) Specimen IVPP V 27,545 in different views. (H-J) Specimen IVPP V 27,546 in different views. (K-N) Specimen IVPP V 27,547 in different views. (O-R) Specimen IVPP V 27,548 in different views. (S-U) Specimen IVPP V 27,549 in different views. (V-Z) Specimen IVPP V 27,550 in different views. Scale bar equals 2 cm.

**Figure 3:**
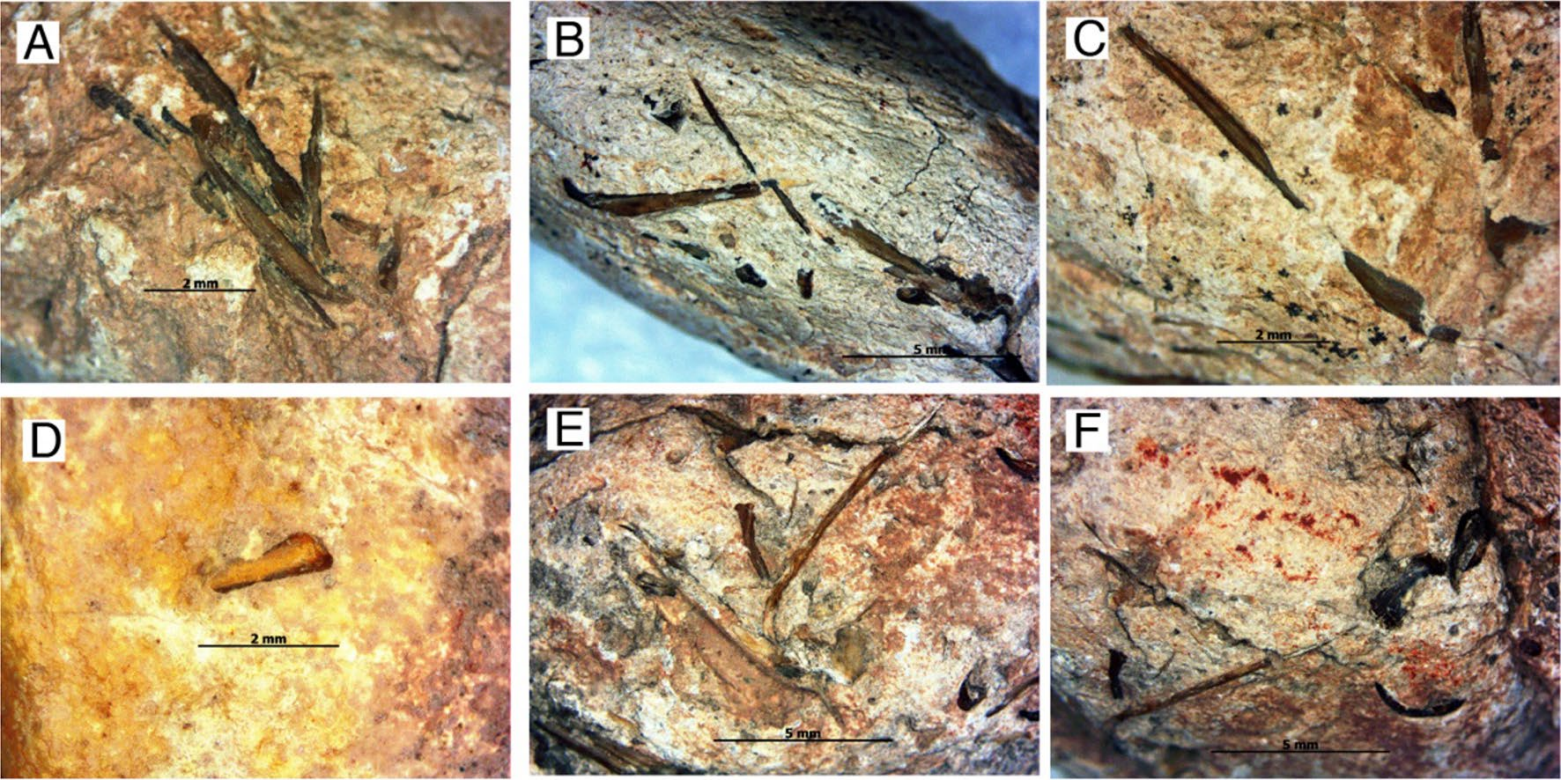
Light microscopy photos of various surface adhesion on the coprolites. Specimen IVPP V 27,545 (A-C). Specimen IVPP V 27,547 (D). Specimen IVPP V 27,544 (E–F). Scale bars as indicated.

- **Holotype:** IVPP V 27,544, coprolite (Fig. 2A–C).
- **Etymology:** For the Tsagan-Tsab formation, which yielded the holotype.
- **Type locality:** Tatal, western Mongolia.
- **Type horizon:** Tsagan-Tsab formation.
- **Referred specimen:** IVPP V-27546 (Fig. 2H–J), IVPP V-27547 (Fig. 2K–N).
- **Description:** *Hyronocoprus tsagantsabensis* was identified through an incomplete holotype and two incomplete referred specimens. These three specimens (Fig. 2A–C, H–J and K–N) are up to 47.79 mm in length, and can reach to a maximum width of 28.34 mm and second width of 25 mm. There are at least three wide coils that are clearly-developed for specimen IVPP V-27544 and IVPP V-27546. Initially, IVPP V 27,546 was thought to be a thyphlosole^9^ morphology. However, with further examinations, we noticed the occurrence of disentanglement at the posterior end, which points to the fact that the actual length of the specimen is unknown. In addition, the ends of all specimens are slightly tapered.
- **Discussion:** *H*. *tsagantsabensis* can be incorrectly classified as heteropolar coprolites due to their incompleteness. A typical morphology for heteropolar coprolites is identified by their tight coils at one end and their exposed flap edge, which is termed ‘lip’ on the anterior end^7^. The coils in referred specimens IVPP V-27544 and IVPP V-27546 are rather spaced. The exposed edge in the referred specimen IVPP V-27547 is not recognised as a flap edge, but rather a rough broken edge of an amphipolar coprolite. On the other hand, the ‘lip’ structure forms an open anterior ending for heteropolar coprolites, which have not been seen in any of our specimens. *H*. *tsagantsabensis* has a familiar morphology as *H. amphipola* (see^51^, Fig. 3I–J), but they vary in size and geologically, as well as geographically. All specimens are considered smooth with inclusions. We proposed P1 for IVPP V 27,544 and IVPP V 27,547, and P2 for IVPP V 27,546.

*Hyronocoprus hunti*, ichnosp. nov., Rummy et al., 2021.

- **Holotype:** IVPP V 27,550, coprolite (Fig. 2V–Z).
- **Etymology:** Dedicated to Adrian P. Hunt, who named the genus in 2005, followed with his major contribution in vertebrate coprolite studies by developing crucial and comprehensive notions and terms.
- **Type locality:** Tatal, western Mongolia.
- **Type horizon:** Tsagan-Tsab Formation.
- **Diagnosis:** *H. hunti* is distinctively large in size. It is generally four times longer than the holotype of *H. amphipola* (see^51^, Fig. 3A–B, specimen NMMNH P-46507) and twice of *H. tsagantsabensis* (IVPP V 27,544).
- **Description:** IVPP V 27,550 (Fig. 2V–Z) is elongated and well preserved with a cross section of a flattened ovoid (Fig. 2Z). It is 94.1 mm long, 30.48 mm in maximum width and 26.87 mm in secondary width. The coprolite has six tight shallow coils that are not sharply separated. One end was slightly broken, but the other end has an acute spot. The surface is smooth with visible cracks and borings.
- **Discussion:** *H. hunti* is similar to some of the *H. amphipola* variance, such as having tight coils and is elongated in shape (see^51^, Fig. 3A–B, specimen NMMNH P-46507). Also, some were seen to have six coils (see^51^, Fig. 3G–H, specimen NMMNH P-46502. We eliminated the fact that it could have belonged to a Mosasaur, due to its smooth surface texture and non-segmented lateral view. We proposed P1 for IVPP V 27,550.

**Notes:**

1. Chin^52^ indicates that large animals can produce small feces, but small animals can’t produce big feces. Thus, deducing the richness of biodiversity of Tsagan-Tsab fauna members, since they are substantially massive than any other spiral coprolite locality members. The existent of a large spiral coprolite producer should be taken critically in order to understand the ecosystem of Tatal during lower Cretaceous.
2. Thus far, *H. hunti* could be the largest spiral amphipolar coprolite found, especially in Mesozoic era.

***Megakalocoprus, ichnogen. nov.***

- **Type ichnospecies:** *Megakalocoprus barremianensis* Rummy et al., 2021.
- **Included ichnospecies:** Known only from the type ichnospecies.
- **Etymology:** From the Latin *mega* (big) and from the Greek *kalos* (rope), in reference to the appearance of an uncoiled rope and *kopros* (dung).
- **Distribution:** Lower Cretaceous of western Mongolia.
- **Diagnosis:** They are distinctively an amphipolar coprolite, as described by Hunt & Lucas^7^. The spirals are spaced along the longitudinal section of the specimen and are not concentrated at one end. *Megakalocoprus* is similar to *Kalocoprus* (see^53^, Fig. 1DD–EE), in the manner of having the same number of coils, with three situated along the lateral length and both differing from *Hyronocoprus* (see^51^, Fig. 3A–B). In particular, there are more than four spirals in lateral view that lack tapering ends. Also, the spirals of *Megakalocoprus* are seen to be separated by a deep sulcus, thus, giving an “unwound” appearances. It has been found that *Megakalocoprus* is three times larger than *Kalacoprus*.
- **Discussion:** The morphology of *Megakalocoprus* is almost identical to *Kalacoprus* and *Hyronocoprus*, as all three of them are distinguished by deep sulci spiral coils. They can be differentiated by the number of coils. Meanwhile, *Megakalocoprus* and *Kalacoprus* are discern by a comparison of their large size.

*Megakalocoprus barremianensis*, ichnosp. nov.

- **Holotype:** IVPP V 27,549, coprolite (Fig. 2S–U).
- **Etymology:** For the Barremian age of lower Cretaceous, which belonged to the holotype.
- **Type locality:** Tatal, western Mongolia.
- **Type horizon:** Tsagan-Tsab Formation.
- **Distribution:** As for ichnogenus.
- **Referred specimen:** IVPP V-27548 (Fig. 2O–R).
- **Description:** Coils on IVPP V 27,549 (Fig. 2S–U) prominently showed similar width in lateral view. Due to their incompleteness, they have an antero-posterior length of more than 49.86 mm. The widest dimension measured at 23.8 mm and has a secondary width of 20 mm. It also has one end that was flattened while the other hand has some damages, which does not affect the prediction of its total coils. There are at least 3 coils in a preserved state and does not extend above 4 coils.
- **Discussion:** The referred specimen IVPP V 27,548 (Fig. 2O–R) is completely preserved without any damages. It has a rough surface with a length of 47.86 mm and a maximum width of 18.61 mm, while its secondary width is 16.59 mm. Coils are clear but not sharply formed as the holotype specimen, which could be due to mucus covering the layer during defecation and quick burial. The middle coil is slightly narrow than the end coil. One end is tapered, while the other end is flattened. Both specimens are similar in the number of coils. We suggest Phase 1 for IVPP V 27,548, and Phase 2 for IVPP V 27,549. Notably, the largest specimen of *Kalocoprus* is 18.8 mm long, while the holotype specimen of *Megakalacoprus* is at least 49.86 mm long.

***Scrollocoprus, ichogen. nov.***

**Figure 4:**
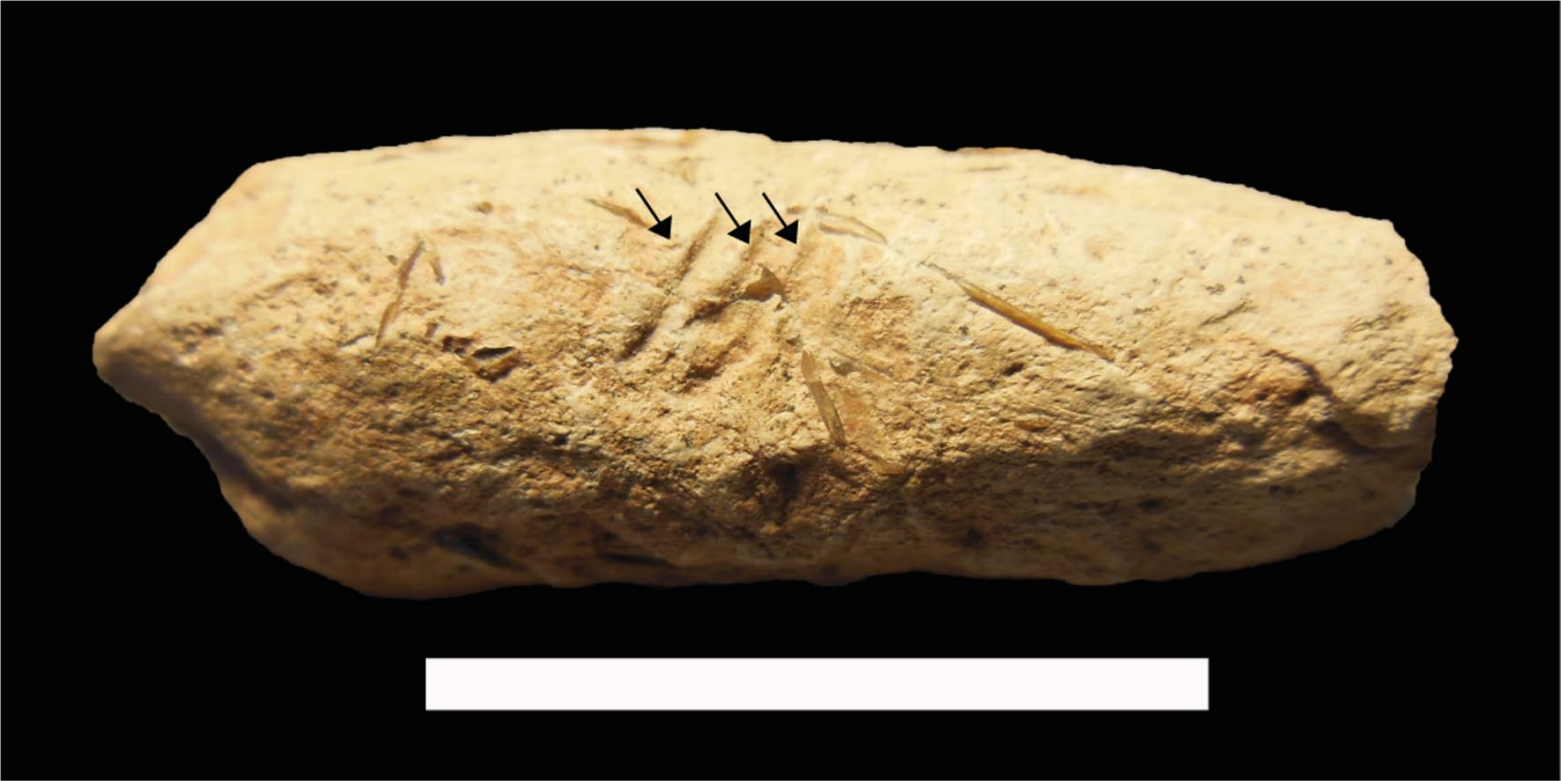
Photo showing external surface of specimen IVPP V 27,545. One side of the specimen has traces of bioerosional scars. Arrows indicates to the furrows. Scale bar equals 2 cm.

**Figure 5:**
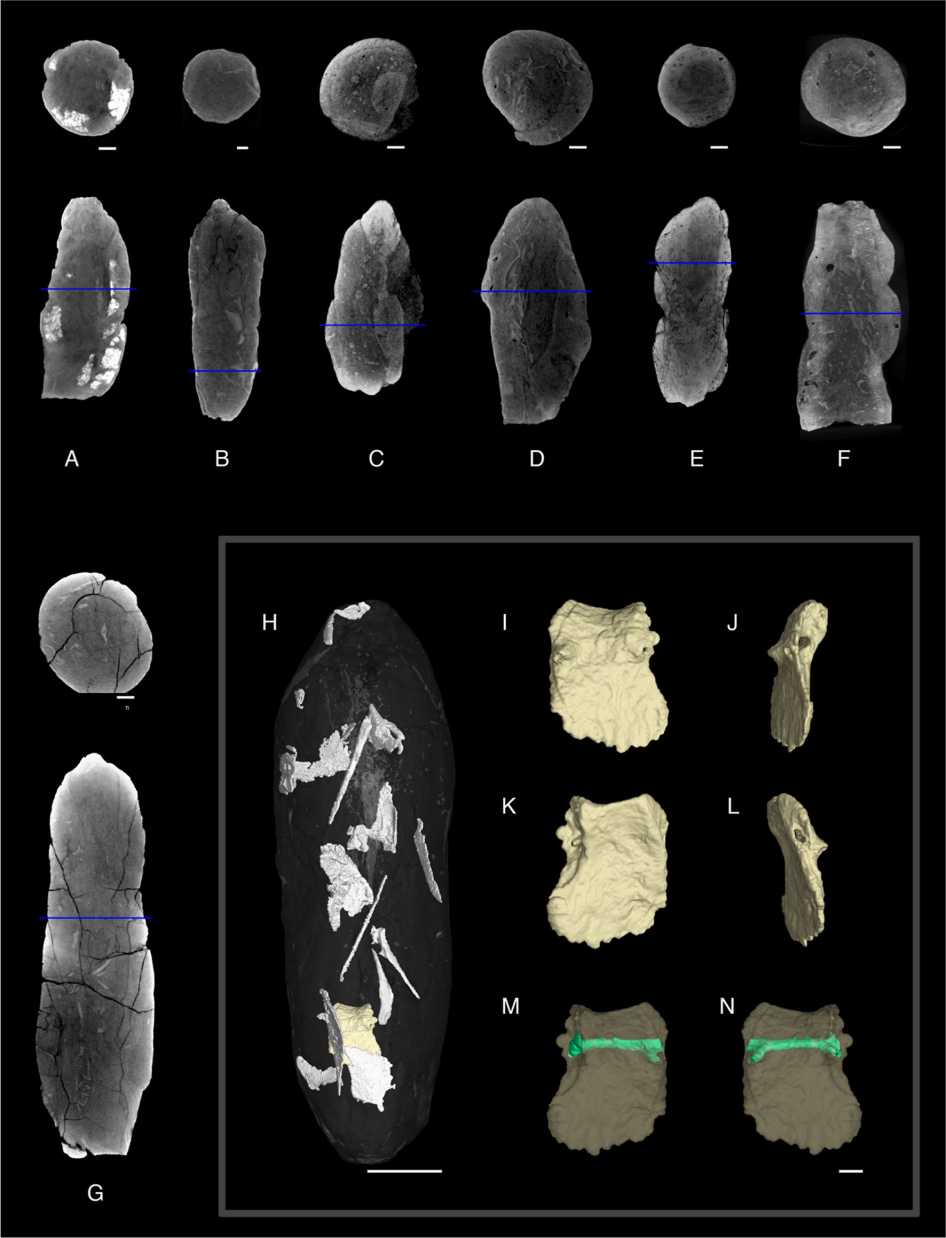
Diagrams showing CT scans of the Tatal’s coprolites. (**A**) to (**G**), each showing a cross and lateral section of all 7 specimens (in order of the specimen numbers, IVPP V 27,544 to IVPP V 27,550). Blue line indicates the area where the cross section was made. (**H**) shows the reconstruction drawing on the bone inclusions in specimen IVPP V 27,545. Structure in yellow indicate the infraorbital bone. (**I**) to (**L**) indicates the infraorbital bone in different angle. (**M**) and (**N**) shows the sensory canal of the infraorbital bone. Scale bars are as following: (**A**) 3500 µm; (**B**) 1500 µm; (**C**) 3500 µm; (**D**) 3500 µm; (**E**) 3500 µm; (**F**) 3500 µm; (**G**) 3500 µm; (**H**) 5000 µm; (**I**) to (**N**) 700 µm.

**Figure 6:**
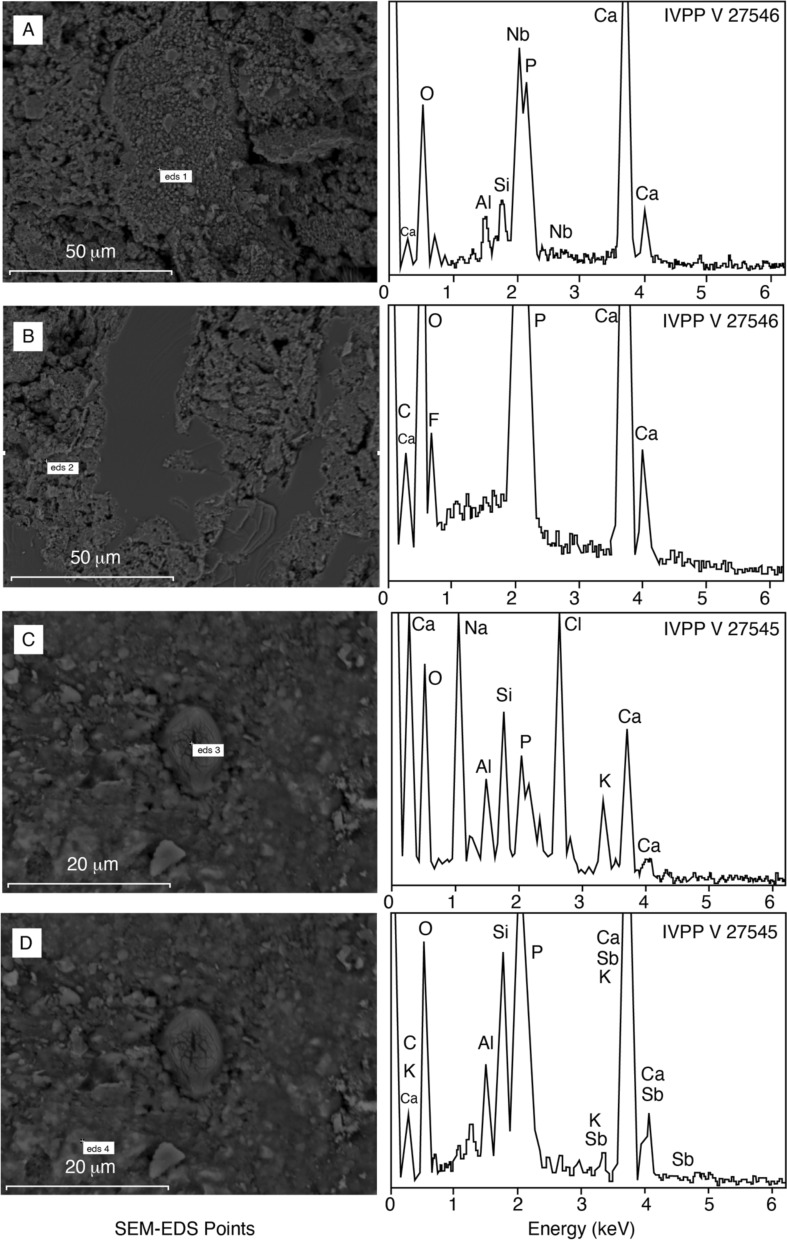
Diagrams showing SEM–EDS analyses results. (**A**) and (**B**) belongs to specimen IVPP V 27,546, while (**C**) and (**D**) to specimen IVPP V 27,545.

**Figure 7:**
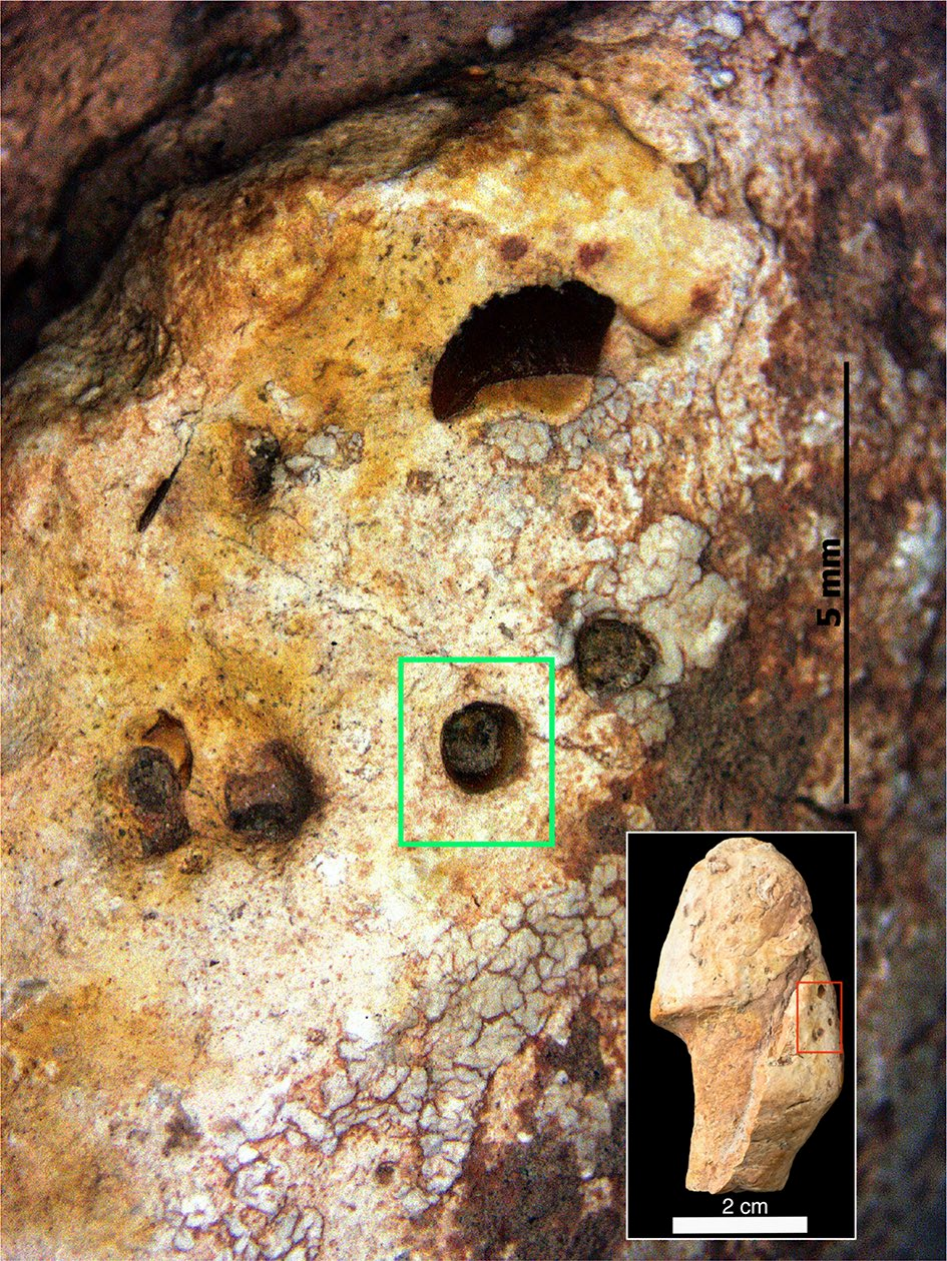
Light microscopy photo of borings on specimen IVPP V 27,547. Box in green shows true burrow while the rest are pseudo-borrows. Inset shows the position of the borings on the coprolite. Scales as indicated.

- **Type species:** *Scrollocoprus tatalensis*, Rummy et al., 2021.
- **Included species:** Known only from the type ichnospecies.
- **Etymology:** *Scroll* from the scroll-liked shape of the coprolite and *kopros* (dung).
- **Distribution:** Lower Cretaceous of Western Mongolia.
- **Diagnosis:** Small, anisopolar, cylindrical rod-like coprolite. It differs from the *Tikicopros* (see^18^, Figs. 4, 5, 6, and 7) and *Bibliocoprus* (see^51^, Fig. 1QQ–SS), in the manner of having tipped posterior end and rounded anterior. Possesses shallow linear or straight free edge that was almost worn out. Also, thin coil that is barely visible on the pointed tip can be seen. More significantly. *Scrollocoprus* differs from *Eucoprus*^7^ , to which *Eucoprus* is perfectly cylindrical in shape, while *Scrollocoprus* has one end that is wider than the other.
- **Discussion:** *Scrollocoprus* is the only scroll coprolite that was found from the Tsagan-Tsab Formation. Thus, making its discovery a noteworthy contribution towards understanding the fauna of Tatal during lower Cretaceous period.

*Scrollocoprus tatalensis*, ichnosp. nov.

- **Holotype:** IVPP V 27,545, coprolite (Fig. 2D–G).
- **Type locality:** Tatal, western Mongolia.
- **Type horizon:** Tsagan-Tsab Formation.
- **Etymology:** Name after the locality, Tatal where the type specimen originates.
- **Distribution:** As for ichnogenus.
- **Description:** IVPP V 27,545 (Fig. 2D–G) is 36.8 mm long, 13 mm in maximum width and 11.52 mm in secondary width. It is a complete coprolite with a rounded cross section. One end is slightly tapered in a rounded shape, as compared to the other end that is tipped.Visible clusters of bone fragments and fish scales can be seen on its surface.
- **Discussion:** At present, this ichnospecies is specifically known to be from the Tsagan-Tsab Formation of western Mongolia. Thus far, this is considered to be the second findings of Mesozoic era’s scroll coprolites, just after the *Tikicopros* of Tiki Formation, India^18^.

## Results

### Sizes

As from the measurements, all collected coprolites vary in sizes (Table 1). The smallest and complete specimen is IVPP V 27,545 (Fig. 2D–G), and while IVPP V 27,550 (2 V-Z) is multiple time larger. The maximum length for specimen IVPP V 27,544, IVPP V 27,546, IVPP V 27,547 and IVPP V 27,549 have not been determined due to their incompleteness.

**Table 1:** Biometrical and morphological features of spiral coprolites from Tsagan-Tsab Formation (Lower Cretaceous), Tatal, western Mongolia. Paul Rummy, Kazim Halaclar & He Chen.

Specimen number	Ichnosp	Max. length (mm)	Max. width (mm)	Second width (mm)	Weight (grams)	Phase	Coloration	External features	Internal Features	Morph. ^Type^
IVPP V 27,544	*H. tsagantsabensis*	46.81	22.38	19.76	25	1	8/1, 2 for gley	Bone fragments; tiny holes;	Bone fragments	Spiral
							8/6, hue 7.5 yr 3/6, hue 10 yr			
IVPP V 27,545	*S. tatalensis*	36.80	13	11.52	7	–	8/3, hue 7.5 yr	Bone fragments; bite marks	Bone fragments	Scroll
							8/1, 2 for gley			
IVPP V 27,546	*H. tsagantsabensis*	42.00	24.09	21.56	22	2	8/1, 2 for gley	Bone fragments; tiny holes;	Bone fragments; borings	Spiral
							7/6, hue 5 yr 5/8, hue 5 yr			
IVPP V 27,547	*H. tsagantsabensis*	47.79	28.34	25.00	30	1	8/1, 1 for gley	Bone fragments; borings	Bone fragments; borings	Spiral
							8/6, hue 10 yr 6/8, 2.5 yr			
IVPP V 27,548	*M. barremianensis*	47.86	18.61	16.59	19	1	7/6, hue 5 yr	Bone fragments	Bone fragments; borings	Spiral
							2.5/3, hue 7.5 yr			
IVPP V 27,549	*M. barremianensis*	49.86	23.85	20.00	33	2	8/1, 2 for gley	Bone fragments	Bone fragments; borings	Spiral
							8/4, 2.5 yr 4/6, hue 10 yr			
IVPP V 27,550	*H. hunti*	94.10	30.48	26.87	97	1	8/2, 2 for gley	Bone fragments; borings; cracks	Bone fragments	Spiral
							8/4, 2.5 yr			

### Surface adhesion and marks

All specimens contained some degree of bone fragments and rhomboidal-shaped ganoid scales adhered to the coprolite surfaces (Fig. 3). Additionally, all specimens have smooth surfaces with little abrasion. The inner coil lines of specimen IVPP V 27,549 adhered with a matrix of red clay with silt (Fig. 2S–U). Only specimen IVPP V 27,550 has been seen with concentric cracks (Fig. 2V–Z). Bite marks have also been found on specimen IVPP V 27,545, in which these traces were short, parallel, shallow and isolated. They have been formed from 3 furrows of roughly 3.8 mm long and 0.3 mm deep (Fig. 4).

### Inclusions

Through CT scans and surface observation, we noticed that all specimens contained bone fragments and scales of varying degrees (Fig. 5). We were unable to identify the bones in detail for specimen IVPP V 27,544, IVPP V 27,546, IVPP V 27,547, IVPP V 27,548, IVPP V 27,549 and IVPP V 27,550, as they were excessive in amount and extremely fragmentary. On the contrary, for specimen IVPP V 27,545, we noticed a rather complete bone structure, such as the ribs and a segment of an infraorbital (Fig. 5H–N). SEM photograph from one random point of specimen IVPP V 27,545 yielded results of the existents of pollen grain (Fig. 6C).

### Borings

Surface borings of invertebrate burrowing can be seen in 2 spiral coprolites, namely IVPP V 27,547 (Fig. 2D–G) and IVPP V 27,550 (Fig. 2V–Z). CT scans revealed that the borings of specimen IVPP V 27,550 did not intrude internally, and it was the same for some of IVPP V 27,547 as well (Fig. 7). Specimens IVPP V 27,546, IVPP V 27,547, IVPP V 27,548 and IVPP V 27,549 are shown to have traces of internal borings (Fig. 5C–F).

### EDS analyses

In this work, in regards to Tatal’s coprolites, the mineral elements were examined by using EDS and the photos were taken with SEM. Analyses was conducted on 2 specimens (IVPP V 27,546 and IVPP V 27,545) with two sample points for each. All 4 samples showed high peaks of calcium and phosphorus. EDS results of specimen IVPP V 27,546 (Fig. 6A–B) and specimen IVPP V 27,545 (Fig. 6C–D) gave similar atomic compositions. They were mainly composed of Ca, P and O and small peaks that belong to Nb, Si, C, K, Fe and Al. We have also described a potential pollen structure under SEM image (Fig. 6C). This possible pollen structure in specimen IVPP V 27,545 (Fig. 6C) showed different atomic elements from the other EDS results, where it contained high peaks of Na and Cl.

### Taphonomy inferences

No signs of abrasion were found on all of the coprolites. Coloration of the coprolites varied, thus, indicating they were buried in different sedimentary conditions. Through the shape of the coprolites, we can deduce that they have indeed spent different amounts of time or phases in water bodies before burial (see above description/discussion). Meanwhile, specimen IVPP V 27,550 showed shallow coil deepness, therefore, this indicates that it was buried rapidly after excretion.

### Discussion and interpretation

There are several pivotal evidences that corroborate to fecal origins of the Tsagan-Tsab Formation material: (1) basic morphology; (2) general shape and size (3) inclusions of the fecal matter; (4) high calcium and phosphorus content; (5) bioerosional scars; (6) borings and cavities; (7) concentric cracks.

The fundamental puzzle in the studies of coprolite is the difficulty in identifying the potential producer, which can be due to their nature and preservation. Also, that includes the methods used to deduce them with their producer, which were done by inferring with various forms of relationship based on stratigraphy and geographical relationships, as well as on neoichnology studies^7,23,54,55^. Such problems similarly arose in our context as well, and the materials were collected from a stratum that were interpreted as lake deposit margins, thus, suggesting an amphibious or aquatic producer. The paleoenvironment correlates with the findings of pterosaur fossils such as the *Noripterus*^44^ or argued as ‘*Phobetor*’^56^, and the diets of these pterosaurs were dependable on the lake environment^57–60^. Above all, and more importantly, that the shape of the coprolite has to be intact in order to represent the shape of the internal intestine of the producer, whereby, anatomically it can lead to a certain biological aspect and digestive system of the organism. Despite these, there are on-going controversies on the origin of the spiral shaped bromalites in regards to whether or not they signify fossilized feces, or they are the cololite that was formed within the colon^6,21,23,61,62^.

Spiral coprolites are producer of an animal with spiral intestine valves to increase the surface area of absorption, to slow down food movement in the bowel to maximise nutrient absorption, which has a significant strategy in surviving uncertain and harsh environment conditions^28,63,64^. Referring to past literature, it is generally agreed upon that the spiral shape is the only distinctively coprolite morphology, whereby it has been regarded as a true coprolite and can be correctly associated to the source animal, such as a range of fishes in particular^6,22,52^. Many primitive bony fishes (except those of teleosts), fresh water sharks (elasmobranches), coelacanths, *Saurichthys*, sturgeons and lungfishes are known to have the spiral valve intestine^51,64–66^. Also, Price^67^ suggested that the amphipolar form could have been derived from palaeoniscoids. Additionally, Romer & Parsons^68^ noted that the spiral valves are secondarily lost in teleost and tetrapods, while Chin^69^ noted a few teleosteans still possessing them.

The spiral coprolites collected for this study are mainly amphipolar in shape and one in scroll. As we know, generally heteropolar spiral coprolite are produced by sharks, which have complex spiral valves^62^. Therefore, we can exclude those in the family of elasmobranches as the potential producers and this can also be supported by the non-marine geological settings of Tsagan-Tsab Formation. But it is also noteworthy to mention that in previous studies, some workers have conducted observations on sharks that were kept in tanks, and were not been able to find any spiral fecal pellets. The reasons given were that the sharks’ eating habits could have changed due to the tank environment, which would have differed from the natural marine environment. Also, modern day sharks are totally unrelated to the ancient Permian pleuracanth sharks^6^. Despite these, evidence of spiral fecal pellet can still be observed in some of the present-day fishes, such as the African lungfish *Protopterus annectans*, the Australian lungfish *Neoceratodus forsteri*, the long-nosed gar *Lepisosteus osseus* and the spotted gar *Lepisosteus oculatus*^6,70–72^. As for scroll coprolites, it is generally known to be produced by animal with longitudinal valves (*valvular voluta*), whereby the valves naturally rolls in upon itself , in a way that it maximises nutrient absorption^8,9,17,18^. Gilmore^17^ in his work mentioned that this type of valve must be primitive than the transverse valve (*valvular spiralis*), which could be a modification of the previous ones. This form is especially known to sharks of carcharhiniforms^73^, and it is evident that it could have been associated with sarcopterygian^53^, as well as anaspid and thelodont agnathans^17^.

In this study, we recognised four new ichnotaxa for all the seven coprolite specimens. Assigning four new ichnotaxa does not conclude that the coprofauna are of four different types of animals. Considering there are two distinct morphologies, which are the amphipolar spiral and scroll, we can deduce that at least two animals can produce these coprolites. But we have to carefully consider that diverse diets at different times for the same animal can often be variable, and soft fecal materials can range disparately after defecation, as well as taphonomy influence^74,75^. Specimen IVPP V 27,550 is remarkably huge and its producer should be a massive animal since large animals could produce small excrement, but small animals would not be able to produce big excrement^52,54^. Moreover, since there are no relevant fossils fauna found in the locality, we were unable to exactly identify the specific producer, rather, we deduced with relevant sources. However, we do know that both amphipolar spiral and scroll coprolites can be attributed to certain types of fishes. As of these, we can conclude that the coprolites were produced by fishes in different sizes. Specimen IVPP V 27,545 differs from the rest by its shape and size, which makes prediction even harder, because it could be produced by either large or smaller animals.

CT scans revealed that bony inclusions are evident in all of the coprolites (Fig. 5). However, except in specimen IVPP V 27,545, the bones in the rest of the coprolites are fragmentary. Specifically, bones in specimen IVPP V 27,545 are rather unaffected by the acidity of the digestive enzyme and these were evident by the presence of clusters of entire bones in the coprolite (Fig. 3A–C), as contrast to the fragmentary bones in the rests of the coprolites. Furthermore, we identified an infraorbital bone of a fish. CT scans revealed that the infraorbital bone has a sensory canal where it branches off at both ends (Fig. 5M–N). With these, we can indicate that the producer of specimen IVPP V 27,545 poorly masticated the prey and also had a rather low gut digestion for food^28,55,76–78^. Through these results, we can infer the digestive strategies of the producers were in correlation with food intake and digestion process, as discussed in Barrios-de Pedro & Buscalioni^77^. Specimen IVPP V 27,545 might belong to the first type of digestive strategy, whereby the producer has limited food processing in the mouth and the food stays in the digestive system for a short period of time. This strategy is regarded to be efficient in conditions where food sources are abundant and the nourishment levels are sufficient^79^. The rest of the coprolites possibly belong to the second digestive strategy, as the bone content is fragmentary. This suggest the producer might have limited mastication with improved digestive assimilation and longer gut time to favour better absorptions of nutrients^55,80–83^. The third type of digestive strategy does not imply in our study. It is also noteworthy to mention that the quantity of the inclusions is not correlated to the size of the coprolite, rather, it is dependable on the above-mentioned biological variables^28,84^.

Carnivorous coprolites are normally composed of calcium phosphate and other organic matter, but it is important to be aware that the initial compositions are usually altered during fossilization processes^33^. Meanwhile, the excretion of herbivores is generally lacking in phosphates and their fossilization are mostly dependable of the mineral enrichment^85^. Through the morphological shape, the density of bone and scale inclusions on the surface from the CT scans, we can directly assume that these coprolites are inevitably produced by carnivorous organisms. Despite that, we still conducted SEM–EDS tests on two specimens, IVPP V 27,546 and specimen IVPP V 27,545 (Fig. 6), in order to determine its mineral content, and to prove them as a valid coprolite material because we were not able to compare these materials to any attached locality matrix at the time the study. The reason for that was because the specimens were collected almost two decades ago and they were very well-kept in the archives throughout these years. As predicted, all 4 samples gave higher content of Ca and P, thus, there is no doubt that they are indeed fossilized fecal materials. Also, in regards to the SEM–EDS on specimen IVPP V 27,545 (Fig. 6C–D), when randomly pointed to a particular structure, it yielded unusual results from the rest, in which the EDS peaks are composed of Na and Cl. At the same time, the SEM image potentially showed a pollen grain like structure. Hollocher and Hollocher^86^ documented a pollen image by using SEM, which brings our potential pollen image (Fig. 6C) dimensionally compatible with their sample. Although specimen IVPP V 27,545 is produced by an unidentified carnivorous vertebrate, it is common for carnivore coprolites to have plant remains within them. Also, it is known that spores and pollens are exceptionally well preserved within the encasement of calcium phosphate, which inhibits sporopollenin degradation^87^. Various reasons can be inferred for the presence of the pollen in specimen IVPP V 27,545, to which it could either be by accident or by preying on an herbivorous animal. Furthermore, it could also be through the adhesion on the excrement when the fecal is still fresh^88^. Pollens are in fact valuable information provider for paleoenvironment reconstruction, as well as for understanding the vegetation state of a particular era^87,89–92^. Hence, further palynology analyses are needed for future work.

EDS mineral composition and coprolite coloration can be correlated to a certain degree, in which it could also explain depositional origin^27^. Most of the Tatal’s coprolites are pink-whitish in color, which is highly associated with the presence of calcium through its carnivorous diets^93–96^. The dark colors can also be due to the presence of iron or it could also be due to complete phosphatisation^23,27^. However, a large part of the colorations was influenced by diagenesis^27,28^.

Traces of burrows are evident on the surface of specimen IVPP V 27,547 and IVPP V 27,550, but CT scans revealed internal traces burrowing did occur in specimen IVPP V 27,546, IVPP V 27,547, IVPP V 27,548 and IVPP V 27,549 (Fig. 5). Since not all possible burrows were dug-in, we gave the term ‘pseudo-burrow’ on those burrows that were abandoned in the early stages. For example, on all of the burrow traces in specimen IVPP V 27,547, only one traces showed burrowing holes, while the rest did not form a hole. While those specimens with internals, but without any traces on the outer surface, this can be explained by taphonomy processes, whereby the outer surface is covered with sedimentary and non-differentiable. It was reported in Tapanila et al.^97^, that marine bivalves are potential makers of the burrows in coprolites by expanding the diameter of the hole as they dig in, although Milàn, Rasmussen & Bonde^98^, reported a contradictory example, where the holes were indeed constant in diameter. In our study, we couldn't determine if the holes were constantly in diameter or not. Numerous tiny holes were visible on all of the coprolites surface, as well as within it, and these were most probably caused by gases within the fecal matters. These holes can be called as microvoids or ‘degassing holes’, which contain gases trapped during digestion^74,99,100^. Microvoids are quickly filled with water when fecal matter is excreted from the animal body, thus making the fecal becoming heavy and sinking to the lake floor^74^.

A series of three parallel furrows or bioerosional scars were evident on the surface of specimen IVPP V 27,545 (Fig. 3). Those lines only occurred once without any repetition on the rest of the surface. The information from these furrows were insufficient to deduce any potential biters, as widely discussed in the work of Godfrey & Palmer^101^, Godfrey & Smith^102^, Dentzien-Dias et al.^103^, and Collareta et al.^104^. On the other hand, deducing from the dented surface on the bitten marks, we predicted that the marks were most probably made by the biting pressures from the fish mandibles, which may indicate coprophagous behavior. The biting could have happened on the lake floor just before sedimentary deposition. Since the bitten marks are on the surface, this probably suggests unintentional scavenging and was eventually aborted during food search.

In general, coprolites can be transported from the original place through various modes^25^ and this can be evident by the traces of abrasion^51,65^. However, in Tatal’s coprolites, there were little or almost no marks of abrasion. Yet again, this supports our hypothesis that these coprolites were excrements in shallow waters, such as in the lake banks with little turbulence and current, where the fecal matter was dropped *in-situ* after excrement. As stated in previous literature^105,106^, radial and concentric cracks are also evident on the surface of specimen IVPP V 27,550, therefore, these indicate that the coprolite was excreted on a very shallow environment where the water body was vastly evaporated and left for subaerial exposure before embedment. This phenomenon caused the coprolite to dehydrate through the cracking, and shrinking occurred in a low magnitude process while retaining its overall shape^27,54,107^. Previous authors have also discussed that the cracks could possibly be due to synaeresis under certain conditions^27,54,108^.

It has been frequently reported in records that almost all spiral coprolite fossilization from various Phenerozoic ages have occurred in low-energy shallow marine environments^54^. Feces that are being excreted in this humid environment have a higher chance of preservation due to the rapid burial, as well as on the acidity level of the water bodies^5,7,109–111^. There are also several crucial factors that are involved in fecal fossilization. Among them, one of the most important criteria includes the content and composition of the fecal matter, and those of carnivorous diets tend to form coprolites than those who consumed an herbivorous diet^75^. As mentioned in Dentzien-Dias et al.^111^, there are three main stages involved in a coprolite taphonomy history, which include stages before final burial, after the final burial and after exposure. In accordance to this, we introduced the usage of phases to discuss the spiral coprolites morphologies in this study (see material and methods). The phase concept of spiral coprolites disentanglement has been widely discussed in early days by various workers^6,22,70^. Coprolite specimen IVPP V 27,544 and IVPP V 27,547 are considered as Phase 1, as the coils are not deep, and this can be explained as during excrement, there’s a mucosal membrane covering the surface of the fecal matter and embedment occurring rapidly, thus retaining most of its surface structure. Although there are signs of disentanglement, we predict that the uncoiling on the surface was not by natural processes, but has been caused by a breakage after on. Both of these two coprolites could have been large in actual size. Similar explanations can be given to specimens IVPP V 27,548 and IVPP V 27,550, whereby the coils are shallow, thus, classifying them as to had occurred in Phase 1. We classify specimen IVPP V 27,546 and IVPP V 27,549 as Phase 2, in which the spaces between the coils of IVPP V 27,546 were slightly separated and in IVPP V 27,549, they were strongly separated. Both of these specimens could have spent more time in water bodies before burial. Specimen IVPP V 27,545 does not provide any external information in regards of phases approach because of its non-spiral morphology. While it is also worthwhile to mention that none of them have spent sufficient time in the water bodies in order to possess the Phase 3 structure. Through these, we can also conclude that smaller coprolites are much complete while bigger coprolites tend to easily break-off. However, having mentioned that, the preservation of specimen IVPP V 27,550 is indeed valuable.

Through the above morphological points, we predict that the amphipolar spiral coprolites could have belonged to groups of either prehistoric lungfishes or Acipenseriformes (sturgeon and paddlefish). Another aim of this work is to portray the existence of possible prey-predation relationships from the collected coprolites. In order to narrow down the identity of the potential producer and possibly the prey, we looked into some related fauna list from past literature. Geological settings have indicated that the Lower Cretaceous Tsagan-Tsab formation is not only recorded in the area of Tatal, but also in other regions of Mongolia as well^36^. There are two possibilities on the deduced prey and predator, they are either of Asipenceriformes—Lycopteriformes relationship or Asipenceriformes—Pholidophoriformes relationship. We suggest Pholidophoriformes as a much potential prey than the Lycopteriformes in the Tsagan-Tsab Formation, and the reasons will be explained thoroughly. As for the producer, we knew that Asipenceriformes are largely known from the *Lycoptera-Peipiaosteus* (Asipenceriformes) Fauna or the “Jehol Fauna”, as these assemblages of fishes were not only abundant in the Lower Cretaceous Yixian Formation of northeastern China, but also widely distributed over the region of eastern Siberia, Mongolia, northern China and northern Korea^112^. It is also noteworthy to mention that the Tsagan-Tsab formations and the Yixian formation were similar in geological age. In the same context, Jakolev^35^ described *Stichopterus popovi* (Asipenceriformes) and recorded amphipolar spiral coprolites from the Aptian lacustrine of Gurvan-Eren Formation of Mongolia , a locality that is close to Tatal. Although there are differences in the geological period of Tsagan-Tsab and Gurvan-Eren Formation, it is highly possible that Asipenceriformes existed in these areas. Furthermore, Asipenceriformes are shown to have spiral valves^113^, and this can be further proven with the work of Capasso^64^ on *Peipiaosteus pani*, thus, contributing to the morphology of the spiral coprolites. With these, we strongly suggest that the amphipolar spiral coprolites of Tsagan-Tsab Formation and for Gurvan-Eren Formation to belong to Asipenceriformes. As for prey, we know from existing literature that there is a close relationship between Asipenceriformes and *Lycoptera,* as evident in the name *Lycoptera-Peipiaosteus* Fauna. Yondon et al.^36^ reported *Lycoptera middendorfii*, a form of small freshwater Teleost fish from the Eastern Gobi—Tsagan-Tsab formation. But, it was clearly mentioned that Bon-Tsagan/Bon-Chagan (Fig. 1) is the westernmost locality of *Lycoptera* in Mongolia^114^. Another fact that was taken into account for the possible prey is the shape of the scales found in the inclusions, whereby Lycoptera are known for their cycloid shaped scales, while the ones in our specimens are more towards rhomboidal-shaped ganoid scales. These facts crucially eliminate the possibilities of *Lycoptera* for the Tsagan-Tsab fauna. With this, we further examined Jakolev^35^′s works and discovered the species that he described, *Gurvanichthys mongoliensis* (Pholidophoriformes) from the Gurvan-Eren Formation has rhomboidal-shaped ganoid scales. The size, shape of the scale and the nature of this fish fits well as a prey for the *Stichopterus popovi* (Asipenceriformes). Through these interpretations, we can possibly infer that the spiral coprolites in our study might have belonged to Asipenceriformes and Pholidophoriformes as the prey, which could further affirm the occurrence of prey-predator inter-relationship in the Lower Cretaceous of Tsagan-Tsab Formation.

As for the sole scroll coprolite in this study, we do not intend to further deduce any detailed possibilities. Based on other works, chondricthyans origins or a sarcopterygian for scroll coprolites were suggested^18,53^,but such deduction is difficult to be purported in our studies as there is a lack of such fossil materials in the locality and surrounding localities. The chances of the underived producer to be a sarcopterygian is much higher than to be a chondricthyan, mainly due to its geological settings. The discovery of the single scroll coprolite can be a window opening to many paleontological questions for Tsagan-Tsab Formation.

## Conclusions

This study significantly contributes to the first detailed study of coprolites from the Mesozoic of Mongolia. Specifically, we recognised four new coprolite ichnotaxa, such as: *Hyronocoprus tsagantsabensis* and *Hyronocoprus hunti*, to which both are ichnosp. nov.; followed by *Megakalocoprus barremianensis* and *Scrollocoprus tatalensis*, where both are ichnogen. et ichnosp. nov. *Hyronocoprus tsagantsabensis*, *Hyronocoprus hunti* and *Megakalocoprus barremianensis* are composed of amphipolar spiral coprolites, while *Scrollocoprus tatalensis* is scroll in shape. Generally, through the SEM–EDS analyses and CT scans, we can conclude that all the studied coprolites have been produced by carnivorous organisms with piscivorous diet. *Scrollocoprus tatalensis* might be omnivorous consisting of animal and plant diets, as bony fish bones and pollen grain were evident, or it could have eaten preys with herbivorous diet. All coprolites were in different sizes, inferring the producers were of different sized organisms. Additionally, the coloration, desiccation cracks, number of borings, cavities and coils deepness are different, indicating that these coprolites are buried under different taphonomy conditions. The producer of *Hyronocoprus tsagantsabensis*, *Hyronocoprus hunti* and *Megakalocoprus barremianensis* can be related to Asipenceriformes, while for *Scrollocoprus tatalensis*, we were unable to specifically link to any particular fish, but it could possibly be of sarcopterygian origins. In addition, we predicted that the prey is from the Order of Pholidophoriformes. The study also shows that the ecology from where the coprolites were retrieved were once abundant in fish fauna. On a concluding note, a comprehensive future fossils’ studies and field excavation on the Tsagan-Tsab Formation is necessary to understand its paleoecology and intraspecies relationship.

### Materials and methods

The coprolites from the Tsagan-Tsab formation that are described in this study consist of 7 specimens (IVPP V 27,544, IVPP V 27,545, IVPP V 27,546, IVPP V 27,547, IVPP V 27,548, IVPP V 27,549, IVPP V 27,550), in which 3 of them are in complete forms. They were collected *in-situ* by a senior researcher of IVPP (X. Wang) during The Mongol Highland International Dinosaur Project in 1998. All of them came from the same locality, together with other fossil faunas, especially the pterosaurs^44^. The specimens are currently being housed at the Institute of Vertebrate Paleontology and Paleoanthropology, Beijing.

### Terminology

The terminology of this paper follows Hunt & Lucas^7^. Five distinct morphotypes can be distinguished within the collected material. The term spiral coprolite is mainly divided into heteropolar, where the whorls or coils are concentrated at one end of the coprolite; while those of amphipolar, are recognized by the spiral which are spaced along the length of the specimen (see^5,22,25^). Another category of coprolite in this study can be termed as rod-like or cylindrical elongated (see^7^, Figs. 1 and 6). The term isopolar is referred to coprolite specimens with ends that are identical while anisopolar are for ends that are different in shape^54^. The definition of total length and coil length follows McAllister^62^. The definition of width follows Larkin, Alexander & Lewis^74^ and Halaclar^115^, where two width measurements were taken, one at the widest diameter and another at 90 degree to the first width. The coprolites were measured using the aid of a vernier caliper to the nearest millimeter by eye. Coloration is based on Munsell soil color chart^116^. Measurements, weight and general characteristics of the 7 specimens are summarized in Table 1.

In this study, we adapted the coil loosening approach to discuss the period of the excrement in the water bodies from the time of excrement to burial. This biological aspect was noted by some workers in the past. In Dean^70^, Williams^6^ and Jain^22^, they observed that these excrements, when deposited in water bodies, tends to uncoil like ribbon like by hourly. In this paper, we noted this process as phases to describe the morphology of the spiral coprolites. With this, we propose 3 phases in order to explain the period of the uncoiling, as explained by the aforementioned authors. Those include: Phase 1—Early phase of deposition, where all coils remain intact; Phase 2—Several hours after deposition, which some coils start to disentangle; Phase 3—After 24 h, where most coils have already loosened.

### Scanning electron microscopy

We conducted the scanning electron microscopy (SEM) coupled with energy-dispersive X-ray spectroscopy (EDS) on two specimens where the samples were easily obtained. For this, a tiny piece of sample was required by breaking it from the coprolite tip. The specimens were then attached to a stub and coated with gold. The least damaging approach was considered in this process, which explains the reason on why only two samples were considered, and not all seven.

### Computed tomography

A non-destructive technique using Computed Tomography (CT) scanning was used in examining the content and borings in the coprolites as well the production of a 3D model (Supplemental video). Seven specimens of coprolites (IVPP V 27,544 to IVPP V 27,550) were scanned using the 225 kV micro-computerized tomography (developed by the Institute of High Energy Physics, CAS) at the Key Laboratory of Vertebrate Evolution and Human Origins, CAS. The specimens of IVPP V 27,544, IVPP V 27,546, IVPP V 27,547, IVPP V 27,548, IVPP V 27,548 and IVPP V 27,550 were scanned with beam energy of 160 kV and a flux of 120 μA at a resolution of 63.00 μm per pixel, and IVPP V 27,545 were scanned with beam energy of 130 kV and a flux of 150 μA at a resolution of 19.13 μm per pixel, using a 360° rotation with a step size of 0.5°. A total of 720 projections were reconstructed in a 2048*2048 matrix of 1536 slices using a two-dimensional reconstruction software developed by the Institute of High Energy Physics, CAS. All of the segmentation and the rendering of the CT scanning data were processed by using VG Studio Max 3.0 (Volume Graphics, Heidelberg, Germany).

### Photography and drawings

Each specimen was photographed and edited with Adobe Photoshop CS6 to remove backgrounds, and drawings were completed by using Adobe Illustrator CS6.

## Disclosures

Natural Science Foundation of China, Grant No. 41688103. Strategic Priority Research Program of the Chinese Academy of Sciences, Grant No. XDB26000000.

## Supplementary Information

Supplementary Information

## Data Availability

All data generated or analysed during this study are included in this published article (and its Supplementary Information files).

## Abbreviations

**CAS**: Chinese Academy of Sciences
**PGI MAS**: Institute of Paleontology and Geology, Mongolian Academy of Sciences, Ulaanbaatar, Mongolia
**IVPP**: Institute of Vertebrate Paleontology and Paleoanthropology, Chinese Academy of Sciences, Beijing, China

## References

[1] Lister, M. *Letters and divers other mixt discourses in natural philosophy*. New York: Printed by J. White for the author (1683).

[2] Duffin CJ (2012). The earliest published records of coprolites. N. M. Mus. Nat. Hist. Sci. Bull..

[3] Mantell, G. A. *The Fossils of the South Downs Or*: Illustrations of the Geology of Sussex, Lupton Relfe (1822).

[4] Buckland W (1829). On the discovery of coprolites, or fossil faeces, in the Lias at Lyme Regis, and in other formations. Trans. Geol. Soc. Lond. Ser..

[5] Neumayer L (1904). Die Koprolithen des Perms von Texas. Palaeontographica (1846–1933).

[6] Williams, M. E. *The Origin of Spiral Coprolites. Paleontological Contributions*. The University of Kansas, Paper 59 (1972).

[7] Hunt A, Lucas S (2012). Descriptive terminology of coprolites and Recent feces. N. M. Mus. Nat. Hist. Sci. Bull..

[8] Owen, R. *On the anatomy of vertebrates*. Volume I, fishes and reptiles: London, Green and Co., 650 p. (1866).

[9] Parker TJV (1880). On the intestinal spiral valve in the genus Raia. Trans. Zool. Soc. London.

[10] McAllister JA (1987). Phylogenetic distribution and morphological reassessment of the intestines of fossil and modern fishes. Zoologishe Jahrbücher Abtheilung für Anatomie und Ontogenie der Thiere.

[11] Steno, N. De Anatome Rajae Epistola. Hafniae. Also published in his De Musculis & Glandulis observationum specimen, etx. Hafniae, 1664.—Maar, 1910, I, No. XVI, pp. 195–207; spiral valve, p. 199 (1664).

[12] Perrault, C. Observations qui ont este faites sur un grand Poisson dissequé dans la Bibliotheque du Roy, *le vingt-quatrieme Juin*, 1667, Paris (1667).

[13] Müller JB (1835). Vergleichende Anatomie der Myxinoiden.

[14] Duméril, A. *Histoire naturelle des Poissons ou ichthylogie generale Paris 2* **vol 1:** 1–720 (1865).

[15] Gudger EW (1950). The history of the discovery (1600–1680) of the spiral valve in the large intestine of Elasmobranchs and a Ganoid. J. Elisha Mitchell Sci. Soc..

[16] Aldridge RJ, Gabbott SE, Siveter LJ, Theron JN (2006). Bromalites from the Soom Shale Lagerstätte (Upper Ordovician) of South Africa: palaeoecological and palaeobiological implications. Palaeontology.

[17] Gilmore B (1992). Scroll coprolites from the Silurian of Ireland and the feeding of early vertebrates. Palaeontology.

[18] Rakshit N, Bhat MS, Mukherjee D, Ray S (2018). First record of Mesozoic scroll coprolites: classification, characteristics, elemental composition and probable producers. Palaeontology.

[19] Dentzien-Dias P, Figueiredo A, Horn B, Cisneros JC, Schultz CL (2012). Paleobiology of a unique vertebrate coprolites concentration from Rio do Rasto Formation (Middle/Upper Permian), Paraná Basin, Brazil. J. S. Am. Earth Sci..

[20] Lea HC (1843). On Coprolites. Am. Philos. Soc. Proc..

[21] Fristsch, A. Miscellenea palaeontologica 1. *Palaeozoica* 1–23 (1907).

[22] Jain SL (1983). Spirally coiled coprolites from the Upper Triassic Maleri Formation India. Palaeontolology.

[23] Duffin CJ (1979). Coprolites: a brief review with reference to specimens from the Rhaetic Bone Beds of England and South Wales. Mercian Geol..

[24] Häntzschel W, El-Baz F, Amstutz GC (1968). Coprolites, an annotated bibliography. Memoirs Geol. Soc. Am..

[25] Hunt, A. P., Chin K. & Lockley, M. G. The palaeobiology of vertebrate coprolites, p. 221–240. In S. K. Donovan (ed.) *The Palaeobiology of Trace Fossil.* Wiley, Chichester, U.K. (1994).

[26] Eriksson ME, Lindgren J, Chin K, Månsby U (2011). Coprolite morphotypes from the Upper Cretaceous of Sweden: novel views on an ancient ecosystem and implications for coprolite taphonomy. Lethaia.

[27] Krause J, Piña C (2012). Reptilian Coprolites In the Eocene of Central Patagonia Argentina. J. Paleontol..

[28] Cueille M, Green E, Duffin CJ, Hildebrandt C, Benton MJ (2020). Fish and crab coprolites from the latest Triassic of the UK: From Buckland to the Mesozoic Marine Revolution. Proc. Geol. Assoc..

[29] Poinar H, *et al.* Molecular Coproscopy: Dung and Diet of the Extinct Ground Sloth *Nothrotheriops shastensis*. *Science* (New York, N.Y.). **281:** 402–6. DOI 10.1126/science.281.5375.402 (1998). 10.1126/science.281.5375.402 9665881

[30] Hofreiter M, Sette D, Poinar HN, Kuch M, Pääbo S (2001). Ancient DNA. Nat. Rev. Genet..

[31] Hollocher T, Chin K, Hollocher K, Kruge M (2001). Bacterial residues in coprolite of herbivorous dinosaurs: role of bacteria in mineralization of feces. Palaios.

[32] Jouy-Avantin F, Debenath A, Moigne AM, Moné H (2003). A standardized method for the description and the study of coprolites. J. Archaeol. Sci..

[33] Sharma N (2005). Fungi in dinosaurian (Isisaurus) coprolites from the Lameta Formation (Maastrichtian) and its reflection on food habit and environment. Micropaleontology.

[34] Hu S, Zhang Q, Zhou C (2012). Fossil coprolites from the Middle Triassic Luoping Biota and ecological implication. J. Earth Sci..

[35] Jakovlev YN (1986). Acipenseriformes in Nasekomye v rannemelovykh ekosistemakh zapadnoy Mongolii. Joint Soviet-Mongolian Palaeontol. Expedit..

[36] Yondon K, Badamgarav D, Yarinpil A, Barsbold R (2000). Cretaceous system in Mongolia and its depositional environments. Develop. Palaeontol. Stratigr..

[37] Vasilev, V. G., Grishin, G.L. & Mokshantsev, K. B. Sovetskaya Geology **2:** 68. (in Russian) (1959).

[38] Martinson, G. G., Sochava, A. V. & Barsbold, R. *Dokl. AN SSSR* 189–1081. (in Russian) (1969).

[39] Shuvalov, V. F. & Trusova, E. K. New data on the stratigraphical position of the late Jurassic and early Cretaceous conchostracans of Mongolia. In Paleontology and biostratigraphy of Mongolia, pp. 182–3. *Transactions of the Joint of Soviet–Mongolian Paleontological Expedition 28* (in Russian) (1976).

[40] Bakhurina, N. N. A pterodactyl from the Lower Cretaceous of Mongolia. *Palaeontologicheskii Zhurnal* **4:** 104–8 (in Russian) (1982).

[41] Shuvalov, V. F. *Palaeogeography and historical development of Mongolian lake systems in the Jurassic and Cretaceous*. In Mesozoic lake basins of Mongolia (ed. Martinson GG), pp. 18–80. Leningrad: Nauka (in Russian) (1982).

[42] Ponomarenko AG (1992). Novye setchatokrylye (Insecta: Neuroptera) iz mezozoya Mongolii. Novye Taksony Iskopaemykh Bespozvonochnykh Mongolii, Sovmestnaya Rossiisko-Mongol'skaya Paleontologicheskaya Ekspeditsiya.

[43] Bakhurina NN, Unwin DM (1995). A survey of pterosaurs from the Jurassic and Cretaceous of the former Soviet Union and Mongolia. Hist. Biol..

[44] Lü J (2009). New material of dsungaripterid pterosaurs (Pterosauria: Pterodactyloidea) from western Mongolia and its palaeoecological implications. Geol. Mag..

[45] Graham SA (2001). Sedimentary record and tectonic implications of late Mesozoic rifting, southeast Mongolia. Geol. Soc. Am. Bull..

[46] Krassilov V (1982). Early Cretaceous flora of Mongolia. Palaeontographica B.

[47] Hasegawa H (2018). Depositional ages and characteristics of Middle-Upper Jurassic and Lower Cretaceous lacustrine deposits in southeastern Mongolia. Island Arc.

[48] Samoilov VS, Benjamini C (1996). Geochemical features of dinosaur remains from the gobi desert South Mongolia. Palaios.

[49] Weishampel, D. B., Dodson, P. & Osmólska, H. *The Dinosauria*. 2nd Edition, 1–880. Berkeley: Universtiy of California Press. ISBN 0–520–24209–2 (2004).

[50] Barrett PM, Butler RJ, Edwards NP, Milner AR (2008). Pterosaur distribution in time and space: an atlas in Flugsaurier: Pterosaur papers in honour of Peter Wellnhofer. Zitteliana B.

[51] Hunt A, Lucas S (2005). The origin of large vertebrate coprolites from the Early Permian of Texas. New Mexico Museum of Nat. History Sci..

[52] Chin, K. *The paleobiological implications of herbivorous dinosaur coprolites: Ichnologic, petrographic, and organic geochemical investigations*. Unpublished Ph.D. Dissertation, University of California, Santa Barbara (1996).

[53] Hunt A, Lucas S, Spielmann J, Cantrell A, Suazo TA (2012). New marine coprofauna from the beeman formation (Late Pennsylvanian: Late Missourian), Sacramento Mountains, New Mexico, USA. N. M. Mus. Nat. Hist. Sci. Bull..

[54] Thulborn RA (1991). Morphology, preservation and palaeobiological significance of dinosaur coprolites. Palaeogeogr. Palaeoclimatol. Palaeoecol..

[55] Luo M. *et al.* Taphonomy and palaeobiology of early Middle Triassic coprolites from the Luoping biota, southwest China: Implications for reconstruction of fossil food webs. *Palaeogeography, Palaeoclimatology, Palaeoecology* 474 DOI 10.1016/j.palaeo.2016.06.001 (2016).

[56] Hone DWE, Jiang S, Xu X (2017). A taxonomic revision of *Noripterus complicidens* and Asian members of the Dsungaripteridae. Geol. Soc. Lond. Spec. Publ..

[57] Wellnhofer, P. *The illustrated encyclopaedia of pterosaurs*. London: Salamander Books, 117–121 (1991).

[58] Unwin DM (2005). The pterosaurs from deep time.

[59] Witton MP (2013). Pterosaurs: natural history, evolution, anatomy.

[60] Chen H (2020). New anatomical information on Dsungaripterus weii Young, 1964 with focus on the palatal region. Peer J.

[61] Stewart, J. D. Enterospirae (fossil intestines) from the Upper Cretaceous Niobrara Formation of western Kansas; in Chorn J, Reavis EA, Stewart JD, Whetstone KN, eds., *Fossil fish studies.* The University of Kansas Paleontological Contributions **89:** 9–16 (1978).

[62] McAllister, J. A. Reevaluation of the formation of spiral coprolites. The University of Kansas. *Paleontological Contributions* **Paper 114:** 12 (1985).

[63] Wetherbee BM, Gruber SH (1993). Absorption efficacy of the lemon shark *Negaprion brevisrostris* at varying rates of energy intake. Copeia.

[64] Capasso, L. First direct evidence of the spiral valve intestine of sturgeons in an exceptionally well preserved early Cretaceous fossil. *Bollettino del Museo Civico di Storia Naturale di Verona* 4323–27 (2019).

[65] Mancuso AC, Marsicano C, Palmap R (2004). Vertebrate coprolites from the Triassic of Argentina (Cuyana Basin). Ameghiniana.

[66] Argyriau T, Clauss M, Maxwell EE, Furrer H, Sànchez-Villagra MR (2016). Exceptional preservation reveals gastrointestinal anatomy and evolution in early actinopterygian fishes. Sci. Rep..

[67] Price P (1927). The coprolitic limestone horizon of the Conernaugh Series in and around Morgantown. West Virginia: Carnegie Museum. Ann..

[68] Romer AS, Parsons TS (1986). The Vertebrate Body.

[69] Chin, K. On the elusive trail of fossil dung in Rosenberg, G.D. and Wolberg, D.L., eds., DinoFest: Proceedings of a Conference for the General Public: Boulder. *The Paleontological Society Special Publication* **7:** 285–294 (1994).

[70] Dean B (1903). Obituary notice of a lungfish. Popular Science Monthly.

[71] Mendoza R, Aguilera C, Rodríguez G, González M, Castro R (2002). Morphophysiological studies on alligator gar (*Atractosteus spatula*) larval development as a basis for their culture and repopulation of their natural habitat. Fish. Biol. Fish..

[72] Hassanpour M, Joss J (2009). Anatomy and histology of the spiral valve Intestine in juvenile Australian lungfish. Neoceratodus forsteri. Open Zool. J..

[73] Friedman V (2012). Vertebrate coprolites from the Lower Eagle Ford Group of north central Texas and their paleoecological significance. NMMNH Bull..

[74] Larkin N, Alexander J, Lewis M (2000). Using experimental studies of recent Faecal material to examine Hyaena coprolites from the west Runton freshwater bed, Norfolk UK. J. Archaeol. Sci..

[75] Chin, K. Analyses of coprolites produced by carnivorous vertebrates. In: Kowalewski, M., Kelley, P.H. (Eds.), The Fossil Record of Predation. *Paleont. Soc. Spec.* **Pap 8:** 43–49 (2002).

[76] Owocki K (2012). Upper Permian vertebrate coprolites from Vyazniki and Gorokhovets, Vyatkian regional stage Russian platform. Palaios.

[77] Barrios-de Pedro S, Buscalioni AD (2018). Scrutinizing Barremian coprolite inclusions to record digestive strategies. Ann. Soc. Geol. Poloniae.

[78] Barrios-de PS (2019). Bones of pycnodontiform fishes (Actinopterygii: Pycnodontiformes) in coprolites from the Barremian fossil site of Las Hoyas (Cuenca, Spain). J. Vertebr. Paleontol..

[79] Cork SJ, Kenagy GJ (1989). Nutritional values of hypogeous fungus for a forest-dwelling ground squirrel. Ecology.

[80] Diefenbach COC (1975). Gastric function in Caiman Crocodilus (Crocodylia Reptilia)-I Rate of gastric digestion and gastric motility as a function of temperature. Compar. Biochem. Physiol..

[81] Davenport J, Grove DJ, Cannon J, Ellis TR, Stables R (1990). Food capture, appetite, digestion rate and efficiency in hatchling and juvenile *Crocodylus porosus*. J. Zool..

[82] Bozinovic F (1993). Fisiología ecológica de la alimentación y digestión en vertebrados: modelos y teorías. Rev. Chil. Hist. Nat..

[83] Stevens CE, Hume ID (1995). Comparative Physiology of Vertebrate Digestive System.

[84] Northwood C (2005). Early Triassic coprolites from Australia and their palaeobiological significance. Palaeontology.

[85] Bradley WH (1946). Coprolites from the Bridger Formation of Wyoming: their composition and microorganisms. Am. J. Sc..

[86] Hollocher K, Hollocher TC (2012). Early processes in the fossilization of terrestrial feces to coprolites, and microstructure preservation. New Mex Museum Nat. Hist. Sci. Bull..

[87] Vajda, V., Pesquero, M. D., Villanueva-Amadoz, U., Lehsten, V. & Alcalá, L. Dietary and environmental implications of Early Cretaceous predatory dinosaur coprolites from Teruel, Spain. *Palaeogeography, Palaeoclimatology, Palaeoecology* 464 DOI 10.1016/j.palaeo.2016.02.036 (2016).

[88] Andrews P, Fernández-Jalvo Y (1998). 101 uses for fossilized faeces. Nature.

[89] Scott L, Fernández-Jalvo Y, Carrión J, Brink J (2003). Preservation and interpretation of pollen in hyaena coprolites: taphonomic observations from Spain and southern Africa. Palaeont. Afr.

[90] Prasad D, Strömberg CAE, Alimohammadian H, Sahni A (2005). Dinosaur coprolites and the early evolution of grasses and grazers. Science.

[91] Bajdek P, Owocki K, Niedźwiedzki G (2014). Putative dicynodont coprolites from the Upper Triassic of Poland. Palaeogeogr. Palaeoclimatol. Palaeoecol..

[92] Zatoń M (2015). Coprolites of late triassic carnivorous vertebrates from poland: an integrative approach. Palaeogeogr. Palaeoclimatol. Palaeoecol..

[93] Edwards PD (1973). Qualitative X-ray diffraction and X-ray fluorescence analysis of some Oligocene coprolites. Contribut. Geol. Univ. Wyoming.

[94] Chame M (2003). Terrestrial mammal feces: a morphometric summary and description. Memo rias do Instituto Oswaldo Cruz.

[95] Nobre P, Carvalho I, Vasconcellos F, Souto P (2008). Feeding behavior of the Gondwanic Crocodylomorpha *Mariliasuchus amarali* from the Upper Cretaceous Bauru Basin Brazil. Gondwana Res..

[96] Pesquero MD (2011). An exceptionally rich hyaena coprolites concentration in the Late Miocene mammal fossil site of La Roma 2 (Teruel, Spain): palaecological and palaeoenvironmental inferences. Palaeogeogr. Palaeoclimatol. Palaeoecol..

[97] Tapanila L, Roberts EM, Bouaré ML, Sissoko F, O’Leary MA (2004). Bivalve borings in phosphatic coprolites and bone, Cretaceous-Paleogene, northeastern Mali. Palaios.

[98] Milàn J, Rasmussen BW, Bonde NC (2012). Coprolites with prey remains and traces from coprophagous organisms from the Lower Cretaceous (Late Berriasian) Jydegaard Formation of Bornholm, Denmark. N. M. Mus. Nat. Hist. Sci. Bull..

[99] Guatier A (1983). Animal life along the prehistoric Nile: the evidence from Saggai I and Geili (Sudan). Origini.

[100] Horwitz LK, Goldberg P (1989). A study of Pleistocene and Holocene hyaena coprolites. J. Archaeol. Sci..

[101] Godfrey SJ, Palmer BT (2015). Gar-bitten coprolite from South Carolina, USA. Ichnos.

[102] Godfrey SJ, Smith J (2010). Shark-bitten vertebrate coprolites from the Miocene of Maryland. Naturwissenschaften.

[103] Dentzien-Dias P, Carrillo-Briceño JD, Francischini H, Sánchez R (2018). Paleoecological and taphonomical aspects of the late miocene vertebrate coprolites (Urumaco Formation) of Venezuela. Palaeogeogr. Palaeoclimatol. Palaeoecol..

[104] Collareta A, Gemelli M, Varola A, Bianucci G (2016). Trace fossils on a trace fossil: a vertebrate-bitten vertebrate coprolite from the Miocene of Italy. Neues Jahrbuch für Geologie und Paläontologie - Abhandlungen.

[105] Zangerl RE, Richardson ES (1963). The paleoecological history of two Pennsylvanian black shales. Fieldiana Geol. Memoirs.

[106] Ash, S. R. 1978. *Geology, Paleontology and Paleoecology of a Late Triassic lake, Western New Mexico* (ed. Ash S. R.) Coprolites, p. 69–73 (Brigham Young University Geology Studies, 1978).

[107] Vogeltanz R (1965). Austrocknungsstrukturen bei koprolithen. Neues Jahrbuch fiir Geologie und Palliontologie Monatshefte.

[108] Broughton PL, Simpson F, Whitaker SH (1978). Late cretaceous coprolites from western Canada. Palaeontology.

[109] Diedrich CG, Felker H (2012). Middle Eocene shark coprolites from the shallow marine and deltaic coasts of the pre-North Sea Basin in central Europe. New Mexico Museum Nat. History Bull..

[110] Stringer GL, King L (2012). Late Eocene shark coprolites from the Yazoo Clay in northeastern Louisiana. N. M. Mus. Nat. Hist. Sci. Bull..

[111] Dentzien-Dias P, Hunt AP, Lucas SG, Francischini H, Gulotta M (2020). Coprolites from shallow marine deposits of the Nanjemoy Formation, Lower Eocene of Virginia, USA. Lethaia.

[112] Chang, M. M. & Miao, D. S. An overview of Mesozoic fishes in Asia. 535–563. In Arratia G, Tintori A. (eds). *Mesozoic fishes 3 – systematics, paleoenvironments and biodiversity*. Verlag Dr Friedrich Pfeil, München (2004).

[113] Doroshov, S. I. & Cech, J. J. Physiology of Sturgeon. *Reference Module in Life Sciences* Elsevier (2017).

[114] Sychevskaya, E. K. Freshwater fishes from the Cretaceous of Siberia and Mongolia. - In: Arratia G & Schultze HP. (org.): *Mesozoic Fishes - Systematics and Fossil Record*. 6–10 July 1997, Buckow, Germany, Abstracts: p. 41 (1997).

[115] Halaclar, K. *Analysis of Middle Miocene Locality of Afyon-Gebeceler Coprolite Findings*. Master Thesis. Ege University (in Turkish with English abstract) (2015).

[116] Munsell Color (Firm). Geological Rock-Color Chart: with Genuine Munsell Color Chips. Grand Rapids (2011).

