# Supplementary material for: The first record of exceptionally-preserved spiral coprolites from the Tsagan-Tsab formation (lower cretaceous), Tatal, western Mongolia
Source: Sci Rep. 2021 Apr 12;11:7891. doi: 10.1038/s41598-021-87090-5 (PMC8041832; doi:10.1038/s41598-021-87090-5)
Supplement: Supplementary file 1 — Supplementary Information [file 41598_2021_87090_MOESM1_ESM.pptx]

## Slide 1
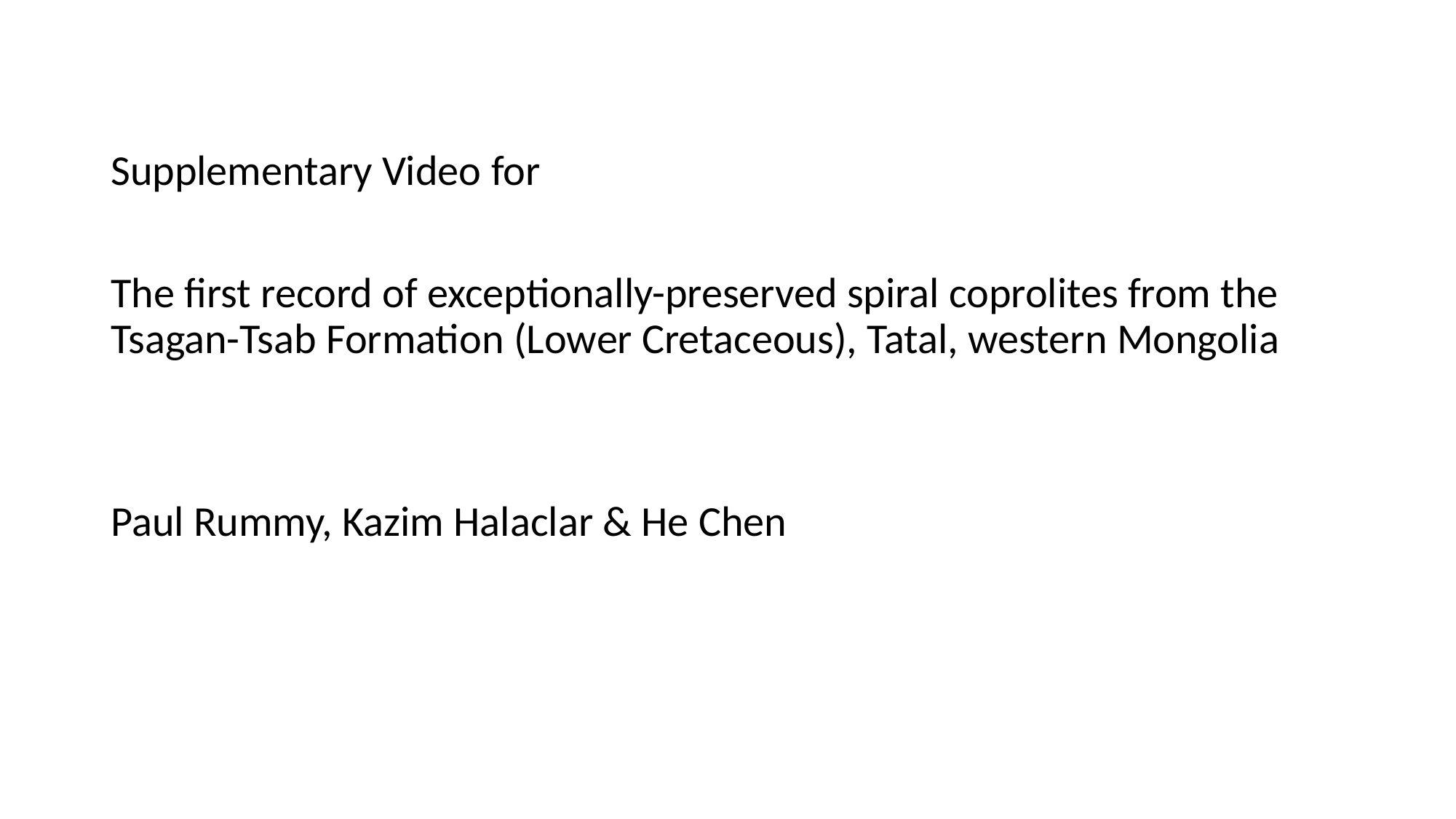

Supplementary Video for
The first record of exceptionally-preserved spiral coprolites from the Tsagan-Tsab Formation (Lower Cretaceous), Tatal, western Mongolia
Paul Rummy, Kazim Halaclar & He Chen

## Slide 2
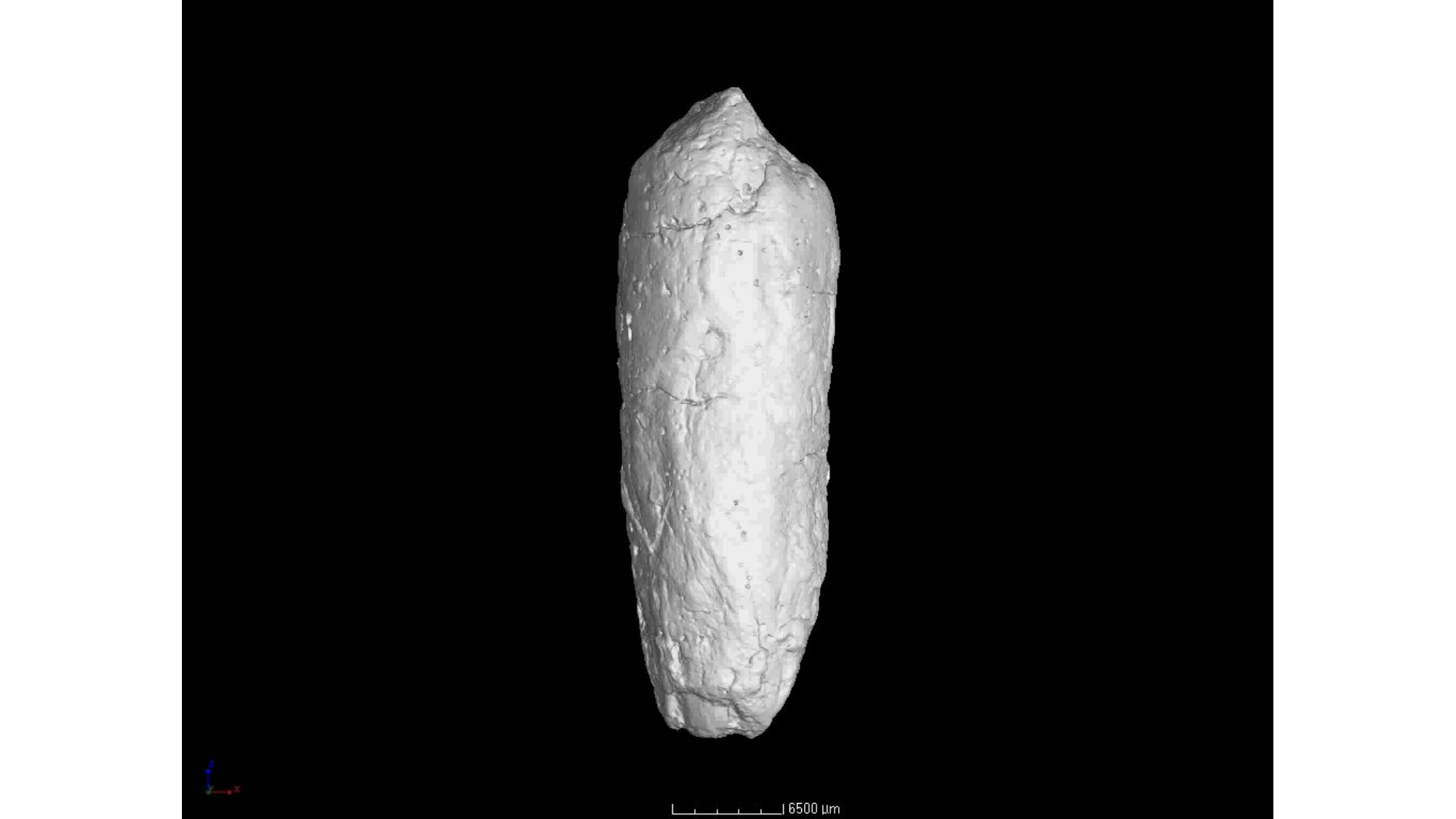

#
